# Diagnosing as autistic people increasingly distant from prototypes lead neither to clinical benefit nor to the advancement of knowledge

**DOI:** 10.1038/s41380-021-01343-3

**Published:** 2021-10-12

**Authors:** Laurent Mottron, Danilo Bzdok

**Affiliations:** 1 Department of Psychiatry and Addictology, University of Montreal, 2900, blvd Edouard-Montpetit, Montreal, QC H3T 1J4 Canada; 2 CIUSSS-NIM, Rivière-des-Prairies Hospital, 7070, Perras Boul, Montreal, QC H1E 1A4 Canada; 3 Department of Biomedical Engineering, McConnell Brain Imaging Centre, Montreal Neurological Institute, Faculty of Medicine, McGill University, Montreal, QC H3A 2B4 Canada; 4 Mila - Quebec Artificial Intelligence Institute, Montreal, QC H2S 3H1 Canada

**Keywords:** Autism spectrum disorders, Neuroscience

## To the Editor:

Since publication of the paper of Mottron and Bzdok [1], we have taken the opportunity to specify the risks/caveats of the claim that the heterogeneity of the so-called “autism spectrum” is forward progress [2]. We did so in responding [3] to the objections by recourse to the familial aggregation of various forms of the phenotype [4], the practical difficulties of operationalizing a judgment of prototypicality [5] and “big data approach” to this question [6]. In a nutshell, we recommend initiating a second round in the recognition-definition-recognition cycle that defines a clinical entity. We suggest separately investigating those who correspond to phenotypically homogeneous categories within the current ‘’autism spectrum”.

This view calls for separating the clinical services and research strategies in terms of their use of diagnosis. Clinical services are based on need, which do not map diagnosis. Research cohorts are recruited and studied to generate knowledge as well as determine to which individuals these insights generalize. Our strategy, which is primarily targeting research, proposes to perform a truncation within individuals ordered by decreasing prototypicality, retaining only the individuals sufficient for the envisioned research endeavor. We suggest first separately studying at least three groups of individuals currently lumped together within the autism spectrum: (a) those with initial language delay, without a non-verbal intellectual disability and identifiable neuro-genetic condition, who show a high level of similarity; (b) those with normal non-verbal and verbal intelligence, without initial language delay which roughly equates with the Asperger of DSM 4, and, (c) “syndromic “ autistics, with a non-verbal intellectual deficiency and measurable neuro-genetic condition or identifiable deleterious mutation.

In contrast, Hobson and Petty [7] view the current heterogeneity of the spectrum as progress that benefits scientific knowledge, societal practices, medical diagnosis, and treatment. The authors provide an example of the benefits resulting, according to them, from the discovery of a “female profile”, distinct from the initial male prototype. This profile is now critically examined [8] and still increases the accepted phenotypic variability of so-called “autism” in women. The absence of a biomarker prevents ascertaining whether such a hypothetical female profile is indeed of the same nature as the male prototype. The result is the undue phagocytosis of other psychiatric conditions with a female sex ratio by autism (for example, borderline personality disorders [9]). The summation of this position across studies gradually equalize the sex ratio of autism diagnoses in meta-analyses [10]. If the classic sex ratio (i.e., 4:1 to 5:1 in male: female diagnoses) was a historical artifact, then, as the commentators suggest, the community would be moving toward greater truth. However, what if the sex ratio previously accepted is mechanistically tied to a relatively homogeneous subgroup of the spectrum? In this case, clinical and basic neuroscientists may end up no longer considering this sex ratio as something that must be explained by neurobiological models. The community will thus have lost information that should contribute to the construction of these models.

Another example of the deleterious effects of a historical trend toward overinclusive diagnoses is the careless assimilation into autism of autistic-like clinical presentations associated with identified genetic mutations or neurodevelopmental syndromes, mostly *de novo*. Such inclusion has been rubberstamped by DSM 5, as the degrees of freedom allowed by the clinical specifier “co-morbidity” subsequently shoehorn these clinical conditions into the autism spectrum. The decision was made *after* the discovery that prototypical forms tend to aggregate in families, with some variations—a concordance that decreases with the amount of genetic material shared with the proband. Discovering that the predisposition to autism is a function of family-specific risk contingencies was immense progress. However, familial predisposition allows for a certain level of clinical heterogeneity, but does not explain it. If, in its early days, the scientific community had included the syndromic presentations as accepted today in the DSM 5 in autism, we would undoubtedly have missed or at least largely underestimated the familial aspect. The scientific community would probably have turned toward de novo mechanisms, which could at first appear to be a decisive discovery accounting for the majority of autism, but which is no longer the case [11].

The distinction between syndromic and non-syndromic autism, even if there are rare ambiguous cases between the two, makes it possible to identify a form of autism. This form affords its own heterogeneity, but is “enriched” in familial mechanisms relative to syndromic autism. However, once syndromic and non-syndromic autism have been distinguished from each other, predisposition should not be confused with the corresponding and clinically relevant condition. We disagree with the notion suggested by Constantino [4], that the distinction between the two is merely “stochastic” and not worthy of scientific interest. We believe that it is necessary to consider a threshold mechanism that, starting from a common genetic predisposition, would result in a relatively similar neurocognitive mode of functioning, with the antagonistic hypothesis that there is no mechanistic unity between homogeneous forms.

Beside research strategies, the commentators defend the clinical benefit to include those phenotypes that are distant from the prototypical form in autism. The benefit of a diagnosis lies in the established, agreed-upon knowledge of a clinical group that can be applied to the individual, including guidance on medical support. However, such a benefit vanishes to the extent that one moves away from the clinical presentation that has given birth to this knowledge. If ‘’female autism” is distinct from predominantly male autism, what benefit will these women have from being given a diagnosis that by definition does not resemble theirs? Currently, the autism spectrum a priori implies that multiple presentations lead to the same diagnosis than familial autism. It should rather make of this disparity a reason to separate these phenotypes and the interventions that benefit to each of them. How can we determine which among subgroups composing the spectrum is informative for the other? In which direction does the external validity flows? It can be decided that all cats are dogs, or vice versa, or that their distinction should be abolished to the benefit of the category “domestic animals”. However, the inclusion of cats and dogs’ behavioral repertoires will be dramatically simplified relative to each of their repertoires considered separately. Such a lumping will result in a loss of information and create an unsolvable puzzle when choosing the direction of external validity between subgroups. Should we train cats to swim, or dogs to climb trees? Probably none—both resist.

Further, Hobson and Petty [7] defend that the recognition of the heterogeneity of the autistic phenotype linked to the presence of a psychiatric comorbidity (e.g.: depression) is a gain in knowledge and leads to better medical care. The insight that autistic people often qualify some psychiatric comorbidities is progress. It should not, however, lead to diagnostic scales that risk of fiercely categorizing certain depressed people as autistic, which is the case with current gold standard tools [12]. The authors recognize, and we agree on this point, that clinical expertise plays a crucial role in limiting the intrinsic overinclusivity of misnamed “diagnostic instruments”. Molloy et al. [13], who they cite, showed the limited ability of such instruments to distinguish ADHD, anxiety and language disorders from what the clinical impression identifies as autism. Indeed, they do not argue in favor of merging all those diagnoses into one category. In suggesting to retain in research only individuals filtered by a clinical opinion, the commenters agree with our position on the superiority in specificity of the recognition of autism over its definition.

Hobson and Petty [7] do not have recourse to convincing arguments that constant increase in prevalence and heterogeneity is progress. They argue that the temporal decrease in effect sizes in autism case-control studies may simply be explained by a tendency for increasing sample sizes. However, Rødgaard et al. [14] found significant negative temporal trends for effect sizes, even when differences in sample size were taken into account. Furthermore, their dismissal of the diminishing effect sizes appears to disregard the well-documented increase in autism prevalence [15, 16] as well as evidence that the symptom threshold for autism diagnoses appears to have decreased over the course of a decade [17], both of which support the interpretation that autism diagnoses are being given increasingly broadly.

The position of Hobson and Petty [7] dominates the scientific community. It is based on the optimistic belief that science progresses in a linear fashion, whereas it actually progresses by leaps and bounds. The science of autism made tremendous progress in the 70s, ruling out irrelevant disease models. We maintain that the progress in the science of autism has moved at glacial speed during the last decade. Decisive progress is still made, but they consist in mostly reporting negative results, by authors who take a radical and critical stance [18]. Autism, as currently operationalized by the “autism spectrum” is not a fruitful subject of study, by bringing together people whose grouping challenge both human and artificial intelligence.

Hobson and Petty [7] rightfully underline that our prototype at hand may escape unambiguous definition. Not only it is hardly definable, but we envision that clinical community is currently more advanced in recognizing that in defining it. The intrinsic fuzziness of the boundaries of a prototype-centered family of forms of life does not prevent the recognition and graduation of a family resemblance. Similarly, the prototype bird does not exist, which does not prevent an excellent reliability in classifying birds by degree of prototypicality [19]. To presume that autism is a “disorder” and cannot be the object of a positive recognition, beyond its contrast with typicality, denies that the living is *organized*. The nature of this organization constitutes the object of both phenomenological and biological psychiatry. The same reasoning would no doubt have also led to the conclusion that Linnaeus’ classification was slowing scientific progress. Would there have been genetics without the preliminary knowledge that forms of life may be classified?
